# Web-based Therapy Plus Support by a Coach in Depressed Patients Referred to Secondary Mental Health Care: Randomized Controlled Trial

**DOI:** 10.2196/mental.8510

**Published:** 2018-01-23

**Authors:** Simon Hatcher, Robyn Whittaker, Murray Patton, Wayne Sylvester Miles, Nicola Ralph, Katharina Kercher, Cynthia Sharon

**Affiliations:** ^1^ Brain and Mind Research Institute Department of Psychiatry The University of Ottawa Ottawa, ON Canada; ^2^ National Institute for Health Innovation The University of Auckland Auckland New Zealand; ^3^ Mental Health and Addiction Services Northland District Health Board Whangarei New Zealand; ^4^ Mental Health Services Waitemata District Health Board Auckland New Zealand; ^5^ Department of Psychological Medicine The University of Auckland Auckland New Zealand

**Keywords:** Internet, major depressive disorder, secondary care, randomized controlled trial, New Zealand

## Abstract

**Background:**

The evidence for the effectiveness of Web-based therapies comes mainly from nonclinical populations, with a few studies in primary care. There is little evidence from patients referred to secondary mental health care with depression. Adherence to Web-based therapies is often poor. One way to increase this is to create a new health service role of a coach to guide people through the therapy.

**Objective:**

This study aimed to test in people referred to secondary care with depression if a Web-based therapy (The Journal) supported by a coach plus usual care would be more effective in reducing depression compared with usual care plus an information leaflet about Web-based resources after 12 weeks.

**Methods:**

We conducted a randomized controlled trial with two parallel arms and a process evaluation that included structured qualitative interviews analyzed using thematic analysis. The coach had a background in occupational therapy. Participants were recruited face-to-face at community mental health centers.

**Results:**

We recruited 63 people into the trial (intervention 35, control 28). There were no statistically significant differences in the change from baseline in Patient Health Questionnaire-9 (PHQ-9) scores at 12 weeks comparing The Journal with usual care (mean change in PHQ-9 score 9.4 in the intervention group and 7.1 in the control group, *t*_41_=1.05, *P*=.30; mean difference=2.3, 95% CI −2.1 to 6.7). People who were offered The Journal attended on average about one less outpatient appointment compared with usual care, although this difference was not statistically significant (intervention mean number of visits 2.8 (SD 5.5) compared with 4.1 (SD 6.7) in the control group, *t*_45_=−0.80, *P*=.43; mean difference=1.3, 95% CI −4.5 to 2.0). The process evaluation found that the mean number of lessons completed in the intervention group was 2.5 (SD=1.9; range=0-6) and the number of contacts with the coach was a mean of 8.1 (SD=4.4; range=0-17). The qualitative interviews highlighted the problem of engaging clinicians in research and their resistance to recruitment: technical difficulties with The Journal, which prevented people logging in easily; difficulty accessing The Journal as it was not available on mobile devices; participants finding some lessons difficult; and participants saying they were too busy to complete the sessions.

**Conclusions:**

The study demonstrated that it is feasible to use a coach in this setting, that people found it helpful, and that it did not conflict with other care that participants were receiving. Future trials need to engage clinicians at an early stage to articulate where Web-based therapies fit into existing clinical pathways; Web-based therapies should be available on mobile devices, and logging in should be easy. The role of the coach should be explored in larger trials.

**Trial Registration:**

Australian New Zealand Clinical Trials Registry (ACTRN): 12613000015741; https://www.anzctr.org.au/Trial/Registration/TrialReview.aspx?id=363351&isReview=true (Archived by WebCite at http://www.webcitation.org/6wEyCc6Ss).

## Introduction

Depression is a common mental disorder [1] with significant impacts on quality of life and with a high social burden [2]. It is also a significant risk factor for suicide [3]. Most treatment of depression occurs in primary care. However, there is limited availability of psychological therapies despite them being recommended as first line treatments for mild to moderate depression. Additionally, access to specialized care is difficult with long waiting lists [4].

There is increasing evidence that Web-based treatments, which deliver psychological therapy based on cognitive behavioral principles, can reduce symptoms of depression [5]. Randomized controlled trials (RCTs) have demonstrated the effectiveness of Web-based cognitive behavior therapy [6], problem solving therapy [7], interpersonal therapy [8], and psychodynamic therapy [9]. However, most RCTs of Web-based therapy have been in “community samples” often recruited from the Internet. These populations are self-selecting, and although their scores on depression rating scales may be comparable to clinical populations, they often differ in comorbidity, duration of symptoms, and impact on everyday activities.

A second problem is whether the Internet interventions should be provided with or without support from a coach or therapist (referred to as guided interventions). The role of the coach is to provide support for the patient progressing through the Web-based therapy, answering both technical questions about how the program works, as well as providing support and encouraging the patient to progress through the program. The computer provides the therapy, whereas the coach provides guidance and advice. To date, coaches have come from a variety of backgrounds including psychology, social work, as well as “technicians” [10,11]. Several systematic reviews have demonstrated that Internet-based interventions for depression provided with human support have effect sizes that are comparable with face-to-face interventions, whereas nonguided interventions have smaller effect sizes [12,13]. However, the evidence is inconsistent with head-to-head comparisons of guided versus unguided interventions showing mixed results. For example, three recent studies on depression showed no significant differences between guided and unguided therapy. The participants were recruited by newspaper or television adverts [14], by a telephone helpline [15], or from US academic internal medicine clinics [16]. In contrast, a head-to-head comparison of guided versus unguided Web-based problem solving therapy in a population of mildly depressed participants recruited via newspaper and television adverts in the Netherlands found that the guided therapy was significantly better than the waiting list control group [17]. These differences could be explained by differences in the Web-based therapy, the population, the type or intensity of support, the different qualifications of the coach, or choice of control group. There have been no trials of guided compared with unguided Web-based therapy in secondary care.

The largest study of guided versus unguided therapy has been the Randomised Evaluation of the Effectiveness, cost-effectiveness and Acceptability of Computerised Therapy (REACT) study [18], which was an RCT of 691 patients with depression in UK primary care. Participants were randomized to treatment as usual, a guided commercial Web-based program “Beating the Blues,” and a guided free Web program “MoodGYM.” The study found no difference in depression outcomes between the three groups. A major criticism of this study is the minimal exposure to the intervention in the Web-based therapy groups; participants only completing a median of 1 or 2 sessions and receiving only 6 min technical support time from the coaches, 5 emails, and almost no text messages (short message service, SMS). About one in 5 participants randomized to Web-based therapy did not access the Web-based programs at all.

The most probable reason that guided Web-based treatments are likely to be effective is that human support increases adherence to the intervention, which results in better outcomes [11]. Unguided Internet interventions have high dropout rates with few people completing the entire course. Mean rates of adherence for unguided interventions are about 26% compared with 72% for guided interventions [19]. The components of effective coaching are unclear; as to date, most of the literature emphasizes the technical aspects of the Internet intervention. It is not clear whether the professional background of the coach makes a difference, what is the optimal frequency of contact, and what the content of the coaching should be.

“The Journal” [20] is a free Web-based program for the self-management of depression developed in New Zealand by the Ministry of Health. It capitalizes on the social marketing appeal of Sir John Kirwan, an ex All Black rugby player who has described his experiences of depression to help destigmatize mental illness. The program is based on the cognitive behavioral techniques of behavioral activation and problem solving. Usage data shows that the depression.org.nz website was visited by 700,000 people in its first year with 20,000 registered with The Journal and 13,000 active users. About 1500 people a month register to start the program, with about three-quarters of people recording significant improvement. There is no data on who uses the program, but peaks in registration coincide with TV adverts promoting the www.depression.org.nz site. Although the program was designed for depression of mild to moderate severity, the evidence shows that nearly a third of people who access the program have more severe depression. However, only one in twenty people who start the program complete all six lessons, and one in ten report no change or a worsening of symptoms. The data do not show who these people are, how to improve the rate of completion, or whether the improvement would have happened without The Journal. The Journal has not been subjected to any clinical trials, and its effectiveness is unproven.

In New Zealand, there is universal health care, with most people having a family doctor. Mental health care is generally provided by community mental health teams based outside hospitals. Each community mental health team provides care to a population of 80,000 to 100,000 people. The teams consist of several disciplines including nurses, psychiatrists, and social workers. People with depression are generally referred by their family doctor.

We report on an RCT and process evaluation of The Journal using a coach compared with a pamphlet that included descriptions of Web-based self-help therapy for depression that participants could choose to use how they liked. We did this in people referred to secondary mental health services with depression. We chose giving an information pamphlet as a control because this seemed like a low cost, low risk alternative that services could easily implement, and we wanted to see if providing guided therapy added anything to this pamphlet. We hypothesized that patients who received guided Web-based therapy would improve quicker and require fewer face-to-face appointments with clinicians than people who received the pamphlet.

## Methods

### Trial Design

The design of the trial was an RCT with two parallel groups.

### Participants

Potential participants were patients attending community mental health centers in Waitemata District Health Board (DHB) who had been referred with a problem of depression or dysthymia. We aimed to involve one community mental health center in an urban area, one in a mixed rural urban area, and the Maori mental health service, Moko. The main exclusion criteria were inability to read and write English or cognitive difficulties, meaning that potential participants would be unable to use a computer. The presence of other comorbid conditions such as anxiety, suicidal thoughts, alcohol, or drug disorders were not exclusion criteria. Potential participants were approached in person face-to-face after triage at the community mental health center to ask for their consent to take part in the trial.

### Interventions

The control group received their normal clinical care. They were also given a pamphlet describing different websites that provide support for people with depression, including The Journal, and told that they could decide for themselves the best way to use the information.

For those participants in the intervention group, the intervention consisted of the following:

1. An invitation to use *The Journal* supported by a coach who provided patients with weekly email, text message, or telephone contact. The coach had a guideline script for each lesson of *The Journal* to reinforce the topic of each lesson, help identify and support patients in their goals, and to coach them in goal setting and the techniques of problem solving. The coach provided a brief summary for the clinicians at each face-to-face appointment, including details of the goals selected by the patients. The coach who had a background in occupational therapy received weekly supervision from an experienced clinician.
2. A brief training program for the clinicians in using *The Journal* with their patients. This familiarized the clinicians in the content of *The Journal* and the clinical concepts embedded in it.
3. A short checklist for clinicians to use to check progress through *The Journal* during scheduled outpatient appointments.

If participants did not have access to a computer at home, one was provided in the mental health center.

### Outcomes

The primary outcome measure was the PHQ-9 [21], a brief self-rating scale for depression that is built into The Journal, measured at 12 weeks (scores of 0-9 on the PHQ-9 indicate minimal or mild depression, 10-14 is moderate depression, 15-19 is moderately severe depression, and 20-27 is severe depression). Secondary outcome measures included the short form-36 (SF-36) [22], the EuroQol-5D (EQ-5D) [23], medication use, time to first outpatient appointment, and the number of outpatient appointments. We administered the rating scales at baseline, after 2 weeks, 6 weeks, and 12 weeks. For measures other than the PHQ-9 in the intervention arm, participants were mailed questionnaires to return in prepaid envelopes or they completed the questionnaires over the phone. Service use within the DHB was obtained from the electronic medical record of each participant.

### Sample Size

Using the PHQ-9 in this patient group based on other studies, we expected the mean pretreatment score to be about 17 with an SD of 4. To detect a difference in score between the two groups of 3 points, an established minimal clinically important difference [24], would need 30 people in each group with a two-sided alpha of .05 and a power of 80%. Allowing for a 25% (20/80) dropout rate, we aimed to recruit 80 participants.

### Randomization

Randomization was by computer, with allocations kept in sequential sealed envelopes at the study base. There were no restrictions. We contacted potential participants by post or telephone. After giving their consent to be in the study, participants were allocated by the study coordinator to one of the groups according to the allocation in the sealed envelopes.

### Blinding

The clinicians were not blind to whether their patients were using The Journal. Research assistants who collected study outcomes were blind to treatment allocation.

### Statistical Methods

Group differences in demographic, pre- and posttreatment measures were analyzed with analysis of variance (ANOVA). We planned to assess changes in participants’ scores from pretreatment to follow-up at 12 weeks by paired sample two-tailed t tests. The summary statistics and the one-way ANOVA were generated in R version 3.0.2 for Windows (R Foundation for Statistical Computing, Vienna, Austria). We planned to use a repeated measure model to account for missing PHQ-9 values. This model analyzed the scores with gender (female and male), ethnicity (Pakeha [non-Maori New Zealanders], Asian, and Maori), age, PHQ-9 (four different times baseline, week 2, week 6, and week 12), group (control and intervention) and their interaction as fixed effects, and PHQ-9 by each patient as a repeated effect. Statistical analysis was conducted using the PROC MIXED procedure in SAS version 9.3 for Windows. A significance level of 0.05 was used in hypothesis testing. Shapiro-Wilk tests were used to assess normal distribution.

We also planned to calculate numbers needed to treat (NNTs) for two measures: (1) the NNT to achieve remission, which we define as a PHQ-9 score of 9 or below at 12 weeks and (2) the NNT to demonstrate a significant reduction in symptoms, which we define as a score of 9 or less or a 50% decrease in individual PHQ-9 scores from baseline to 12-week follow-up. Chi-square was used to test differences in proportions.

### Process Evaluation

We conducted a process evaluation to explore the implementation, receipt, and context of the intervention to help understand the results following the Medical Research Council’s guidelines on assessing complex interventions [25]. We documented the number of lessons completed and the number of email and phone contacts from the coach. We also conducted structured qualitative interviews that asked open-ended questions about participants’ experience of the website, the content of the program, and the experience of having a coach. Interviews were analyzed using thematic analysis using two independent reviewers. The female interviewer was not part of the team that conducted the RCT. We planned to interview a purposive sample of participants with an even gender balance. We would stop interviews once no new themes were identified. We also planned focus groups to interview clinicians in the community mental health teams about their experience of seeing participants who had used The Journal with a coach. The coach provided feedback on her role.

We received signed informed consent from participants, and the study received ethical approval from the New Zealand Central Health and Disability Ethics Committee ref 12/CEN/53.

## Results

### Participants

**Figure 1:**
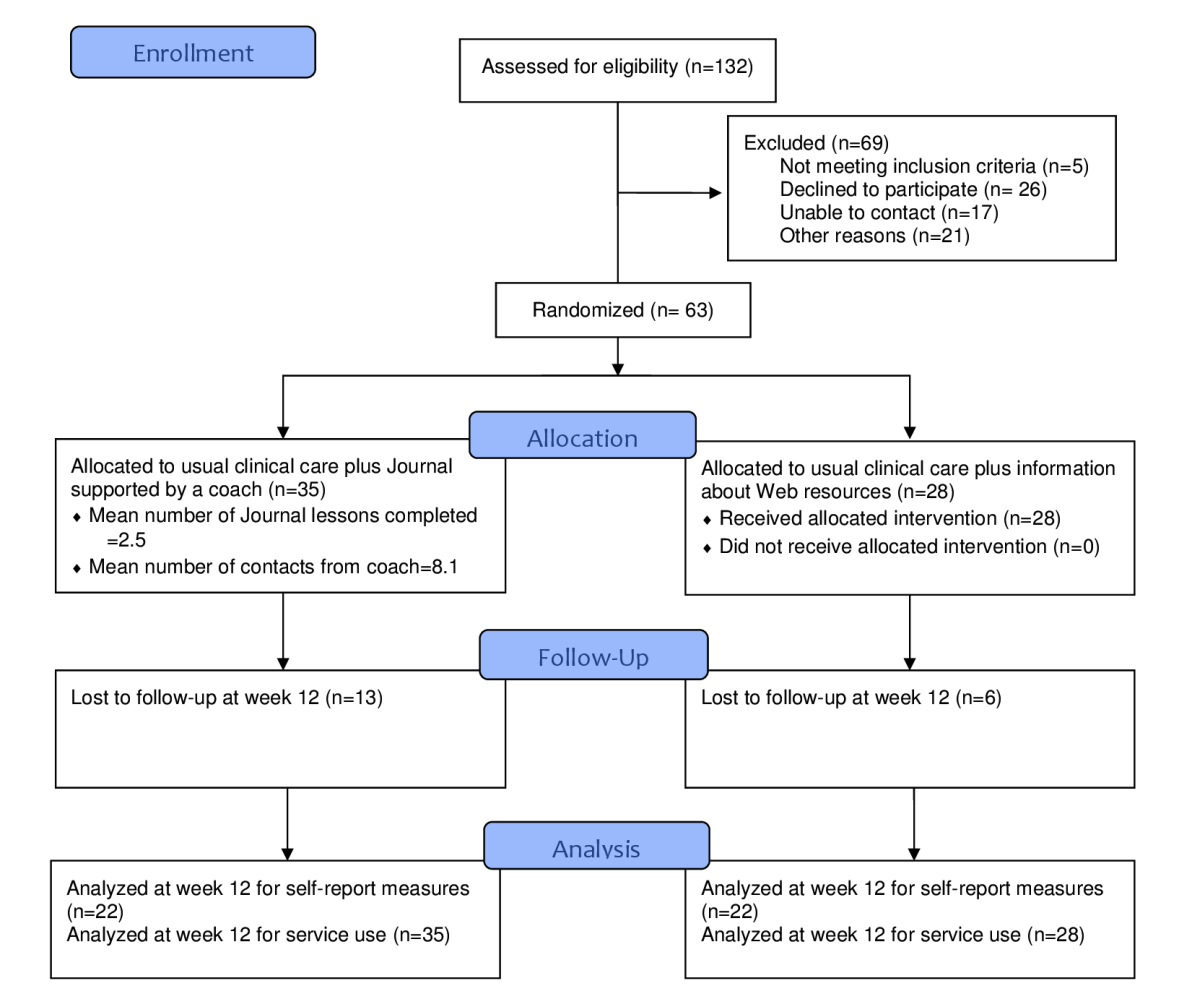
Consolidated Standards of Reporting Trials (CONSORT) flow diagram.

We recruited participants from February to October 2013. Over the 9 months, 132 people were screened, and 63 consented to take part in the trial (47.7% [63/132] of those screened). Of those who declined to consent for the study, 26 preferred not to take part (6 of whom had a private psychotherapist); 17 we could not contact, 5 needed an interpreter, and one had already completed the Journal. Most of the participants (54 of 63) came from two community mental health teams: North One, which covers the southern urban part of the North Shore of Waitemata DHB and Rodney, which covers the more rural northern part of the DHB. Figure 1 shows the flow of participants through the study, and Table 1 describes the baseline data of the participants.

### Primary Outcome

The mean and median PHQ-9 scores were lower in the intervention group than in control at all time points (Table 2). There were more missing values of PHQ-9 scores in the intervention group than in the control group except at baseline. There were high rates of missing scores at 6 and 12 weeks. Shapiro-Wilk tests of normal distribution were not significant apart from the control PHQ-9 scores at week 12 (Shapiro-Wilk statistic 0.91, df=22, *P*=.04).

The one-way ANOVA (Table 2) showed that PHQ-9 scores did not differ significantly between control and intervention groups at any of the time points. Due to the nonnormality of PHQ-9 scores in the control group at week 12, we also tested statistical significance of the difference in mean PHQ-9 scores at week 12 with a Mann-Whitney test. This confirmed that the difference was not statistically significant (Mann-Whitney *U*=193, Z=−1.15, *P*=.25).

The mean change from baseline in PHQ-9 score at 12 weeks was 9.4 (SD 6.7) in the intervention group and 7.1 (7.5) in the control group (*t*_41_=1.05, *P*=.30; mean difference=2.3, 95% CI −2.1 to 6.7).

The repeated measures modeling showed the scores on the PHQ-9 were not statistically significantly associated with gender (*F*_60_=0.65, *P*=.42), ethnicity (*F*_60_=0.74, *P*=.39), age (*F*_60_=0.79, *P*=.46), group (*F*_60_=1.26, *P*=.27), and the interaction between group and PHQ9 (*F*_205_=0.46, *P*=.71). The scores of PHQ-9 differed statistically at the different time points (*F*_186_=40.66, *P*<.001).

**Table 1:** Baseline data.

Variable		Intervention (N=35)	Control (N=28)
**Gender, n**			
	Male	16	13
	Female	19	15
Age in years, mean (range)		43 (21-65)	42 (23-64)
**Ethnicity, n**			
	European New Zealander	21	20
	Maori	2	2
	Other	12	6
**Referral source, n**			
	Primary care	26	14
	Other	9	14
PHQ-9^a^ (SD)		17.1 (5.2)	18.0 (5.7)
SF-36^b^ physical function (SD)		78 (21)	74 (27)
SF-36 social function (SD)		31 (19)	27 (19)
EQ-5D^c^ (SD)		8.0 (1.8)	8.0 (1.7)

^a^PHQ-9: Patient Health Questionnaire-9.

^b^SF-36: short form-36.

^c^EQ-5D: EuroQol-5D.

**Table 2:** Summary scores of Patient Health Questionnaire-9 (PHQ-9) at four time points.

Summary statistic	Baseline		2 weeks		6 weeks		12 weeks	
	Control (N=28)	Intervention (N=35)	Control (N=28)	Intervention (N=35)	Control (N=28)	Intervention (N=35)	Control (N=28)	Intervention (N=35)
Mean (SD)	18.0 (5.2)	17.1 (5.7)	12.4 (6.4)	11.5 (5.2)	11.5 (7.2)	10.1 (5.9)	10.4 (7.7)	7.3 (4.9)
Median	19	17.5	12	11	13	10	7	7
Missing values (%)	1 (3.6)	1 (2.9)	3 (10.7)	6 (17.1)	5 (17.9)	10 (28.6)	6 (21.4)	13 (37.1)
Difference in mean scores (95% CI) and results of one way analysis of variance	0.9 (3.6 to −0) *F*_60_=0.37 *P*=.55		0.9 (4.1 to −2.2) *F*_53_=0.37 *P*=.55		1.4 (5.2 to −2.5) *F*_47_=0.51 *P*=.48		3.1 (7.1 to −0.8) *F*_43_=2.61 *P*=.11	

At 12 weeks, 16 (73%, 16/22) of the intervention group scored 9 or below on the PHQ compared with 12 (55%, 12/22) in the control group (χ^2^=1.57, *P*=.21). To assess a significant reduction in symptoms, we used the last observation carried forward for those participants who had at least two measures on the PHQ-9. This showed that 22 of the intervention group (69%, 22/32) and 15 of the control group (58%, 1/26) had achieved scores of 9 or less or had a 50% improvement in PHQ-9 scores by week 12 (χ^2^=0.76, *P*=.39).

### Secondary Outcomes

Participants in the intervention group attended one fewer outpatient appointments with the mental health teams compared with the control group. This difference was not statistically significant (intervention: n=33, mean number of visits=2.8, SD=5.5 compared with mean=4.1, SD=6.7, n=24 in the control group; *t*_45_=−0.80, *P*=.43; mean difference=1.3, 95% CI −4.5 to 2.0). They also made fewer phone calls to the mental health teams (intervention: n=35, mean=2.4, SD=3.1 compared with mean=2.8, SD=4.1, n=28 in the control group; *t*_61_=−0.50, *P*=.62; mean difference=−0.5, 95% CI −2.3 to 1.4). Participants in the intervention group reported fewer appointments with general practitioners (intervention: n=26, mean number of appointments=1.5, SD=1.2 compared with mean=2.1, SD=2.7, n=22 in the control group; *t*_46_=−0.96, *P*=.35; mean difference=−0.6, 95% CI −1.7 to 0.6).

At 12 weeks, there were no significant differences in mean SF-36 scores between the intervention and control groups (intervention group: n=22, mean SF-36 physical function=87, SD=19; control group: n=22, mean=81, SD=24; *t*_42_=0.9, *P*=.40, 95% CI for difference −7 to 19. Intervention group: n=22, mean SF-36 social function=60, SD=22; control group: n=22, mean=59, SD=30; *t*_42_=0.14, *P*=.90, 95% CI for difference −15 to 17). There were also no significant differences in mean EQ-5D scores at 12 weeks (intervention group: n=22, mean EQ-5D=6.5, SD=1.4; control group: n=22, mean=7.2, SD=1.8; *t*_42_=−1.4, *P*=.16, 95% CI for the difference −1.7 to 0.3).

At the end of the study, 22 out of 26 (85%) people in the intervention group reported they had been prescribed medication compared with 16 out of 22 (73%) in the control group. In the control group, 13 of 22 participants took a mean of 2.4 days off work at 12 weeks compared with 12 of 22 participants in the intervention group who took a mean of 1.5 sick days (*t*_42_=−0.9, *P*=.40, 95% CI for the difference −2.8 to 1.1 days).

### Process Evaluation

The mean number of lessons completed in the intervention group in the 12 weeks was 2.5 (SD=1.9; range=0-6); the number of contacts with the coach was a mean of 8.1 (SD=4.4; range=0-17). Seven people in the intervention group did not complete any of the Journal lessons, although 5 of these did have some contact with the coach. There was no correlation between the number of lessons completed and change in PHQ-9 scores at 12 weeks (Spearman rho, ρ=.1, *P*=.60) or between the number of contacts with the coach and change in PHQ-9 scores at 12 weeks (ρ=.4, *P*=.07).

We conducted nine structured qualitative interviews with 5 male and 4 female participants who had been randomized to The Journal. Interviews took place at a mutually convenient location, which in most cases was a community mental health team base or the person’s place of work. The themes that arose from peoples’ experience of using the website included technical difficulties with access and not being able to view future lessons as barriers to engagement. Furthermore, at the time of the trial, The Journal could not be used fully on a mobile phone or tablet, which participants found limited their use of the program. The participants liked the layout of The Journal, the suggested activities, and the alerts, as illustrated in the following quote:

I liked the one where you had to think about a problem and try and come up with solutions to solve that problem. So chose wanting to spend more quality time with my children and so I found that that really got me thinking about it and to figure out how I could do it.

034;3

Participants found some lessons too long and complicated. They found the coach was helpful for motivation and support, as illustrated in the following quote:

I think that if I had been doing it on my own I would have struggled…but when I got a bit mixed up with it she (E-therapy Coach) was able to say well ok this is what this is and...so I thought the combination of the two (the Journal and coaching) was great.

013;4

However, the process of using a coach was different to a face-to-face appointment, and participants thought education around this would be helpful. Some participants reported avoiding the coach and feeling judged if they felt they had done something “wrong” such as not completing a lesson, as illustrated in the following quote:

One of the lessons I did have a bit of a relapse because she said that I had done it all wrong, and I got quite upset...And so she went away and it was a bit open ended and all I could think of was that I had done it all wrong.

039;6

The feedback from the coach was that some people were not in a “therapeutic space” when accessing help online. When seeing clinicians face-to-face, the act of going prepares individuals to enter a psychological as well as physical space to seek support, explore, open up, and learn. Being online or at home was providing help in a different context that wasn’t necessarily therapeutic. Follow-up by phone was similar as participants often were not available for follow-up when they said they would be or were in places such as supermarkets where coaching conversations were difficult. The coach used email and text messages mainly to check whether people had time to visit the website and to arrange times to call. The coach also found staff in secondary services did not appreciate Internet information and support as part of a skill set and resources they could offer clients.

We conducted two focus groups with clinicians. The main findings were that clinicians wanted to know more about The Journal but thought there were barriers to using it (access to broadband and computers) and that navigating the program was “a bit clunky.” The clinicians also thought it was difficult introducing the program at triage and preferred to use it as an extra resource at discharge from their service.

## Discussion

### Principal Findings

In a trial of usual care plus a guided Web-based therapy compared with usual care plus information about Web-based therapies, in a depressed secondary care population, we found no statistically significant differences in outcomes.

### Limitations

The strength of the study is that it is one of the first trials of a guided therapy compared with unguided therapy in a secondary care setting. It also showed that it was feasible to use a coach in this setting. The main weakness was the trial was underpowered for the outcomes based on self-rating scales because of the large number of dropouts from the study at 12 weeks and because of difficulties recruiting the planned sample size in a clinical setting.

There was also the problem of contamination: (1) the clinicians could use The Journal with patients allocated to the usual care group and (2) The Journal is freely available online, so any participant in the study could access it. There was little we could do to prevent clinicians and patients from using The Journal who were not in the intervention group. However, our experience is that clinicians or patients in secondary care do not widely use The Journal. Furthermore, the problem with contamination is likely to bias the study to showing no-difference (as the control group could use The Journal unguided), so any differences that are found are likely to be more “believable.”

### Comparison With Prior Work

The findings in this study are similar to the REACT study [18], which found no difference between guided and unguided therapies in UK primary care. As in the REACT study, we found low engagement with the Web-based program, although in our study there was greater engagement with the coach.

A problem with The Journal during the study was there were difficulties with ease of access as it was not available on mobile devices and there were problems logging in. The process evaluation identified these as major obstacles, which are similar to problems of losing trust in face-to-face therapy and barriers to access preventing use of services. If patients are to use Web-based therapies as alternatives to face-to-face therapy, then they need to be easily accessible and do what they promise.

This study also indicates that training for patients in the use of a remote coach would be helpful. The training should include explaining the purpose of the coach, state that patients will not be “judged” if they do not complete or miss lessons, what to do if lessons are missed, and the need to be in an appropriate physical place when engaged in coaching. Future studies could include tests of technological ability as a predictor of adherence. Not everyone navigates around the Web-based environment in the same way or with the same level of skill. These tests could include simple timed tasks to see how easily people navigate around Web-based programs on their chosen platform. Additionally, assessments of motivation to complete the program from both the coach and participants could be investigated as predictors of completion of Web-based programs.

### Conclusions

Larger studies in clinical populations in primary and secondary care need to be done. Researchers need to consider when and how Web-based therapies should be used in existing clinical pathways.

## Abbreviations

ANOVA: analysis of variance
DHB: district health board
EQ-5D: EuroQol-5D
NNT: number needed to treat
PHQ-9: Patient Health Questionnaire-9
RCT: randomized controlled trial
SF-36: short form-36

## Appendix

Multimedia Appendix 1 CONSORT-EHEALTH checklist (V 1.6.1).
